# When to Lance the Gums

**Published:** 1873-11

**Authors:** J. L. Smith


### ARTICLE IX.

### *When to Lance the Gums.*

Dr. J. L. Smith says, in his late work on "Diseases of Infancy and Childhood:" "The gum lancet is now much less frequently employed than formerly. It is used more by the ignorant practitioner, who is deficient in the ability to diagnosticate obscure diseases, than by one of intelligence, who can discern more clearly the true pathological state. Its use is more frequent in some countries, as England, under the teaching of great names, than in others, as France, where the highest authorities, as Rilliet and Barthez, discountenance it.

"It is well to bear in mind, as aiding in the elucidation of this subject, the remark made by Trousseau, that the tooth is not released by lancing the gum over the advancing crown. The gum is not rendered tense by pressure of the tooth, as many seem to think, for, if so, the incision would not remain linear, and the edges of the wound would not unite, as they ordinarily do by first intention within a day or two. This speedy healing of the incision, unless the tooth is on the point of protruding, is an important fact, for it shows that the effect of the scarification can only last one or two days. The early repair of the dental follicle is probably conservative so far as the development of the tooth is concerned. It may help us to understand how active, how powerful, the process of absorption is, if we reflect that the roots of the deciduous teeth are more or less absorbed by the advancing second set, without much pain or suffering from the pressure. If the calcareous particles of the teeth are so readily absorbed, what is the foundation for the belief that the fleshy substance of the gum is absorbed with such difficulty? Too much importance has evidently been attached to the supposed tension and resistance of the gum in the process of dentition.

"Follicles in the period of development are especially liable to inflammation. We see this in the follicular sitomatitis and enteritis so common when the buccal and intestinal follicles are in the state of most rapid growth. Does not this law in reference to the follicles hold true of those by which the teeth are formed, so that the period of their enlargement and greatest activity, which corresponds with the growth and protrusion of the teeth, is also the period when they are most liable to congestion and inflammation?

This fact affords a better explanation of the frequency of the so-called laborious or difficult dentition than that it is due to the resistance which dental evolution encounters from the gums. "If there are no symptoms except such as occur directly from the swelling and congestion of the gum, the lancet should seldom be used. The pathological state of the gum which would, without doubt, require its use, is an abscess over the tooth. As to symptoms which are general or referable to other organs, as fever and diarrhœa, the lancet should not be used if the symptoms can be controlled by other safe measures. All co-operating causes should first be removed, when in a large proportion of cases the patient will experience such relief that scarification can be deferred.

"If the state of the infant is such that life is in danger, as in convulsions, or there is danger that the infant will be permanently injured or disabled, as by paralysis, every measure which can possibly give relief should be employed without delay. In these dangerous nervous affections, therefore, the gums, if swollen, should be lanced. I know no accidents of dentition which require prompt scarification except suppurative inflammation of the gums, convulsions, and paralysis. In other cases the operation may be safely postponed till other measures have been employed.—*Med. and Sug. Report.*

